# Resilience as a Mediator Between Workplace Violence and Psychological Well-Being in Hospital Nurses

**DOI:** 10.3390/nursrep15070234

**Published:** 2025-06-26

**Authors:** Mariano García-Izquierdo, María Isabel Soler-Sánchez, José Manuel de Haro García, María Isabel Ríos-Rísquez, Mariano Meseguer-de Pedro

**Affiliations:** 1 Department of Psychiatry and Social Psychology, Faculty of Psychology, University of Murcia, 30100 Murcia, Spain; mgarciai@um.es (M.G.-I.); marianom@um.es (M.M.-d.P.); 2 Department of Business Organisation, Faculty of Economics, University of Miguel Hernández Elche, 03202 Elche, Spain; jharo@umh.es; 3 Hospital Morales Meseguer, 30008 Murcia, Spain; mi.rios@um.es; 4 Department of Nursing, Faculty of Nursing, University of Murcia, 30100 Murcia, Spain

**Keywords:** workplace violence, resilience, psychological well-being, nursing staff, mediational analysis

## Abstract

Workplace violence is a widespread issue affecting hospital nursing staff and significantly undermines their psychological well-being. Such violence originates from various sources, including users, colleagues, and supervisors. Psychological resilience has been linked to more favourable indicators of well-being. **Background/Objectives:** This study aimed to explore how different sources of workplace violence (users, colleagues, and supervisors) are related to psychological well-being and psychological resilience. Additionally, it examines whether resilience is statistically associated with a mediating role in the relationship between source-specific workplace violence and the psychological well-being of hospital nurses. **Methods:** A cross-sectional, multicentre, descriptive, and mediational study was conducted with a sample of 447 hospital nurses. Participants completed a self-administered questionnaire assessing workplace violence from users, colleagues, and supervisors, alongside measures of resilience, psychological well-being, and sociodemographic characteristics. **Results:** Among all reported incidents of workplace violence in the previous year, 69.2% were attributed to users, with verbal abuse (68.7%) being more prevalent than physical aggression (24.1%). Additionally, 37% of nurses reported experiencing violence from colleagues, and 25% from supervisors. Workplace violence from all three sources was significantly associated with both psychological well-being and resilience. Resilience was statistically associated with a mediating role in the relationship between workplace violence and nurses’ psychological health, suggesting a potential mechanism of influence without implying causality. **Conclusions:** The prevalence of workplace violence from users, colleagues, and supervisors among hospital nurses is notably high. Findings indicate that violence from any of these sources is negatively associated with psychological well-being. However, resilience mitigates this impact by reducing psychological distress, positioning it as a crucial personal resource for nurses facing such adversity. These results underscore the need for interventions aimed at developing and strengthening resilience among hospital nursing staff. Moreover, the findings can inform the design of organisational strategies to prevent violence and to promote resilience and well-being within healthcare settings.

## 1. Introduction

Workplace violence against nurses is a global issue of significant magnitude, raising serious concerns due to its detrimental impact on nurses’ health and psychological well-being [1,2]. Workplace violence is any act or threat of physical violence, harassment (unwanted behaviour that affects a person’s dignity), intimidation, or other threatening disruptive behaviour that occurs in the workplace with the intent to abuse or injure the victim. It includes everything from threats and verbal abuse (swearing, yelling, threatening behaviour, non-serious threats, or sexual intimidation) to physical assaults that create an explicit or implicit risk to the health, well-being, and safety of nurses [3]. This workplace violence usually comes from users (patients and their families), colleagues (lateral violence), or supervisors [4,5].

Prevalence rates vary widely across studies, ranging from 25% to 100% for general workplace violence [6,7], between 1% and 87% for lateral violence [8], and up to 80% for supervisor-perpetrated violence [9]. Nurses, particularly those working in hospital settings, are among the most exposed professional groups [10]. Exposure to workplace violence is associated with a heightened risk of anxiety and depression [11], low job satisfaction [12], emotional exhaustion, and an overall deterioration in well-being [13,14]. It can also negatively affect the quality of care provided [15].

In light of this evidence, identifying personal resources that can buffer the psychological effects of workplace violence is of utmost importance. In recent years, resilience has emerged as a crucial personal resource in this context [16].

Psychological resilience refers to the process through which individuals adapt positively in the face of adversity, stress, or trauma. It is a dynamic process that enables nursing staff to effectively cope with stressful, adverse, and traumatic working conditions [16,17].

Although extensive research has focused on the consequences of workplace violence, relatively few studies have examined how individual resources may mitigate its negative psychological effects [18].

The present study conceptualises resilience as a key personal resource within the framework of Hobfoll’s Conservation of Resources (COR) theory [19]. The core proposition of the COR theory is that individuals strive to obtain, retain, foster, and protect those resources they highly value. Personal resources are defined as positive self-evaluations regarding one’s capacity to exert control over and influence the environment successfully. These resources can reduce the perceived demands of the environment and, consequently, the likelihood of negative responses, such as psychological distress. Stressful situations are theorised to arise either when resources are threatened or when there is an actual loss. In this context, workplace violence may represent a threat to personal resources, prompting individuals to mobilise or enhance their resources—in this case, resilience—as a means of mitigating the adverse psychological consequences.

Accordingly, the present study pursued two main objectives. First, to examine how different types of workplace violence—based on their source (users, colleagues, and supervisors)—are associated with psychological well-being and resilience among hospital nurses. Second, and based on prior research suggesting the mediating role of resilience [20,21,22], this study sought to assess whether psychological resilience mediates the relationship between workplace violence and psychological well-being, depending on the origin of the violence.

Based on the reviewed literature and the theoretical assumptions of the COR theory, the following hypotheses were proposed:

**Hypothesis** **1a.** *Workplace violence, perpetrated by users, colleagues, or supervisors, is negatively associated with psychological well-being.*

**Hypothesis** **1b.** *Workplace violence, perpetrated regardless of its source, is negatively associated with nurses’ psychological resilience.*

**Hypothesis** **1c.** *Psychological resilience is positively associated with psychological well-being.*

**Hypothesis** **2.** *Resilience will show an indirect statistical association between workplace violence (from users, colleagues, and supervisors) and psychological well-being.*

This approach offers a more nuanced understanding of the psychological toll of workplace violence and highlights the relevance of targeting resilience as a key protective factor in diverse hospital contexts.

## 2. Materials and Methods

### 2.1. Design and Sample

A cross-sectional survey study was conducted across three public university general hospitals located in Murcia, Spain. The target population comprised registered nurses employed at these institutions between January and March 2023 Nurses who had worked in their current unit for less than six months or who belonged to the hospital’s floating staff (i.e., personnel who work sporadically across various departments) were excluded from this study. A non-probabilistic purposive sampling method was employed. The minimum required sample size was estimated at 323 participants, based on a 5% margin of error, a 95% confidence level, and a target population of 1995 nurses. The questionnaires were distributed in person by members of the research team. Once completed, participants sealed them in blank envelopes and deposited them at a designated collection point, where they were later retrieved by the research team. A total of 642 questionnaires were distributed, of which 447 were returned and deemed valid. The response rate was 69.6%, which is considered acceptable for this type of study. However, no information was available regarding the reasons for non-participation. Of the total sample, 86% were women. The average age was 42.4 years (SD = 10.3; range: 23–65), with a mean of 9.12 years of professional experience (SD = 9.2; range: 6 months to 39 years). Most participants held permanent contracts (58.8%), whereas 36.5% were on temporary contracts. The most common work shifts were rotating shifts (43.6%) and fixed morning shifts (38.6%). Respondents worked in a range of hospital departments typical of large healthcare institutions, including surgery, internal medicine, emergency care, infection control, neurology, and laboratory services.

### 2.2. Instruments

In addition to collecting sociodemographic and occupational information, this study utilised the following validated instruments:

Workplace Violence from Users. Verbal and physical aggression from users and their families was assessed using the Hospital Aggressive Behaviour Scale–Users (HABS-U) [23]. The scale comprises 10 items (e.g., item 9: “Users get angry with me because of delays”), rated on a six-point Likert scale ranging from 1 (never) to 6 (daily). In the present study, the internal consistency was satisfactory (Cronbach’s alpha = 0.86).

Workplace Violence from Coworkers and Supervisors. Violence originating from colleagues and supervisors was measured using the Hospital Aggressive Behaviour Scale–Coworkers–Superiors (HABS-CS) [24]. The coworker subscale contains 10 items (e.g., item 8: “There are coworkers who spread false rumours about me”), and the supervisor subscale consists of 7 items (e.g., item 5: “My supervisor ignores me”). Responses are given on a six-point Likert scale (1 = never to 6 = daily). Internal consistency was high, with Cronbach’s alpha values of 0.91 for coworker-related violence and 0.93 for supervisor-related violence.

Psychological Resilience. Resilience was assessed using the 10-item version of the Connor–Davidson Resilience Scale (CD-RISC-10) [25], validated for Spanish working populations [26]. The scale evaluates an individual’s capacity to adapt to stress and adversity. Items (e.g., item 1: “I am able to adapt when changes occur”) are rated on a five-point Likert scale ranging from 0 (totally disagree) to 4 (totally agree). Total scores are obtained by summing item responses, with higher scores indicating greater resilience. Internal consistency in the current sample was strong (Cronbach’s alpha = 0.89).

Psychological Well-Being. The 28-item version of the General Health Questionnaire (GHQ-28) [27], adapted to the Spanish population [28], was used to measure psychological well-being. Items (e.g., item 10: “Have you felt constantly under strain?”) are rated on a four-point scale (1 = less than usual to 4 = much more than usual). Lower scores on this scale are interpreted as indicators of greater psychological well-being, based on the absence of symptoms. Conversely, higher scores reflect lower psychological well-being (i.e., greater psychological distress). Internal consistency in this study was excellent (Cronbach’s alpha = 0.90).

### 2.3. Data Analysis

Statistical analyses were conducted using SPSS version 28.0 (IBM Corporation, Armonk, NY, USA). The percentage of missing data was low (<5%). No systematic patterns were observed in the missing values with respect to sociodemographic variables or other observable characteristics; therefore, the data were assumed to be missing at random (MAR).

All regression and mediation analyses were adjusted for key sociodemographic variables, including gender, contract type, and years of professional experience, to control for their potential confounding effects. No subgroup analyses were conducted, and the sample was analysed as a whole given the absence of theoretical or empirical justification for stratification.

First, descriptive statistics (means, standard deviations, and Pearson correlation coefficients) were calculated for the primary study variables to examine bivariate relationships between workplace violence (from users, colleagues, and supervisors), psychological resilience, and psychological well-being. The internal consistency of each scale was assessed using Cronbach’s alpha.

Prevalence rates were calculated by considering any score above 1 (“never”) as indicative of having experienced at least one incident of violence from a given source (i.e., a score ≥ 2).

In all mediation models, age, gender, and years of professional experience were included as covariates. These control variables were entered directly into Hayes’ PROCESS macro (Model 4), allowing for adjusted estimation of both direct and indirect effects. This approach reduces the likelihood of spurious effects associated with sociodemographic or occupational characteristics. Indirect effects were estimated using bootstrapping with 10.000 resamples. Effects were considered statistically significant if the bias-corrected 95% confidence interval did not include zero. This non-parametric and robust estimation procedure is widely recommended in health and social sciences research [29].

## 3. Results

Descriptive statistics, including means, standard deviations, and bivariate correlations among the main study variables, are presented in Table 1.

Of all workplace violence incidents reported during the past year, 69.2% were attributed to users, with verbal aggression (68.7%) being more frequent than physical aggression (24.1%). Additionally, 37% of nurses reported having experienced violence from colleagues, and 25% from supervisors.

With regard to psychological well-being, 14.3% of participants reported high levels of psychological distress, whereas 63% demonstrated high psychological resilience.

All forms of workplace violence were positively correlated with each other and with psychological distress and were negatively correlated with resilience. Moreover, resilience exhibited a significant negative correlation with psychological distress.

Independent-sample *t*-tests were conducted to examine potential differences in key variables based on gender and employment contract type. No significant differences were found based on gender (all *p* > 0.10). However, nurses with temporary contracts reported significantly higher resilience scores (t(288.58) = −2.07, *p* = 0.039) and lower psychological distress (GHQ scores; t(243.42) = 2.36, *p* = 0.019) than those with permanent contracts.

Table 2 displays the direct and indirect effects of workplace violence on psychological well-being via resilience. Indirect effects were considered statistically significant when the bias-corrected 95% confidence intervals did not include zero.

As shown in Table 2, resilience was statistically associated with an indirect pathway linking workplace violence to psychological well-being across all sources of violence. Specifically, the indirect effects were statistically significant for violence perpetrated by users (*B* = 0.058, *SE* = 0.014, 95% CI: [0.032, 0.089], *p* < 0.001), supervisors (*B* = 0.066, *SE* = 0.016, 95% CI: [0.038, 0.094], *p* < 0.001), and colleagues (*B* = 0.061, *SE* = 0.015, 95% CI: [0.035, 0.087], *p* < 0.001).

Regarding the direct effects (controlling for the mediator), workplace violence from users (*B* = 0.36, *SE* = 0.07, 95% CI: [0.23, 0.49], *p* < 0.001) and supervisors (*B* = 0.40, *SE* = 0.11, 95% CI: [0.19, 0.61], *p* < 0.001) remained significantly associated with lower psychological well-being. However, the direct effect of violence from colleagues was not statistically significant (*B* = 0.17, *SE* = 0.10, 95% CI: [−0.02, 0.37], *p* > 0.05).

The models accounted for between 6% and 11% of the variance in psychological well-being, depending on the source of violence. In all three models, exposure to workplace violence was significantly associated with lower levels of resilience, which in turn predicted poorer psychological well-being. These findings support the hypothesised mediating role of resilience.

**Figure 1:**
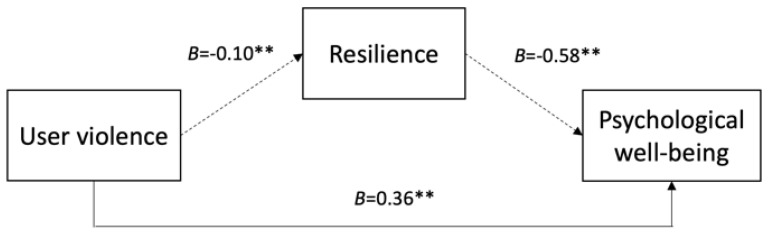
Mediating effect of resilience on the relationship between user violence and psychological well-being. Note. Values shown (B) are unstandardized coefficients with significance indicated (** *p* < 0.01) and 95% confidence intervals (CI). Indirect effect: *B* = 0.058, 95% CI [0.032, 0.089]. Solid lines represent direct effects; dashed lines indicate indirect effects mediated by resilience. Control variables: age, gender, and job tenure.

**Figure 2:**
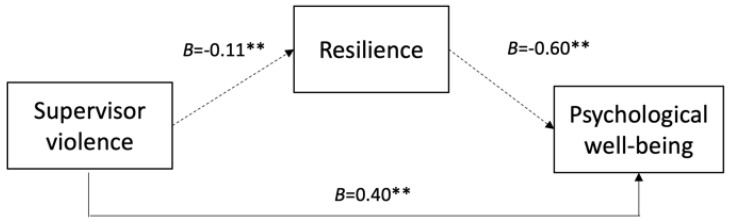
Mediating effect of resilience on the relationship between supervisor violence and psychological well-being. Note. Values shown (B) are unstandardized coefficients with significance indicated (** *p* < 0.01) and 95% confidence intervals (CI). Indirect effect: *B* = 0.066, 95% CI [0.038, 0.094]. Solid lines represent direct effects; dashed lines indicate indirect effects mediated by resilience. Control variables: age, gender, and job tenure.

**Figure 3:**
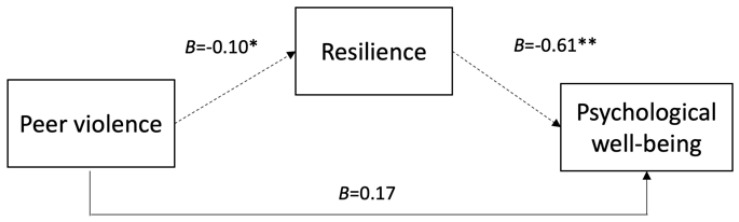
Mediating effect of resilience on the relationship between peer violence and psychological well-being. Note. Values shown (B) are unstandardized coefficients with significance indicated (* *p* < 0.05; ** *p* < 0.01) and 95% confidence intervals (CI). Indirect effect: *B* = 0.061, 95% CI [0.035, 0.087]. Solid lines represent direct effects; dashed lines indicate indirect effects mediated by resilience. Control variables: age, gender, and job tenure.

Below, in Figure 1, Figure 2 and Figure 3, we present the conceptual models illustrating the mediated relationships between the three forms of workplace violence, resilience, and psychological well-being.

## 4. Discussion

The purpose of this study was twofold: first, to examine the relationships between workplace violence from different sources (users, colleagues, and supervisors) and nurses’ psychological well-being and resilience; and second, to assess whether resilience is statistically associated with an indirect relationship between such violence and psychological well-being, without implying causality.

The findings revealed that users were the primary source of reported violent incidents, predominantly verbal rather than physical, followed by colleagues and supervisors. The observed prevalence rates were high and consistent with those reported in the international literature [2,6,7,9]. These findings are also in line with reports suggesting an increase in violence during the COVID-19 pandemic, which may still persist in healthcare settings [13]. In the context of the Spanish national health system, structural factors, such as high patient loads, limited resources, and extensive bureaucratic demands, may contribute to increased stress and elevate the risk of workplace violence, particularly in hospital units with high patient turnover. Although national and regional regulations mandate the implementation of workplace violence prevention protocols, the practical application of such measures is often inconsistent and, in many cases, reactive rather than preventive. This institutional context may contribute to feelings of helplessness among healthcare professionals and exacerbate the psychological impact of workplace violence.

Hypothesis 1a was supported, as workplace violence was negatively associated with psychological well-being. Hypothesis 1b was also confirmed, indicating that violence from all three sources was significantly associated with lower levels of resilience. In addition, Hypothesis 1c was supported, as resilience was positively associated with psychological well-being. These results are consistent with prior research conducted in healthcare settings [30,31].

Hypothesis 2 was also supported. Resilience partially mediated the relationship between violence perpetrated by users and supervisors and nurses’ psychological well-being. However, in the case of coworker-related violence, the direct effect on well-being was not statistically significant, whereas the indirect path through resilience was. This statistical pattern suggests full mediation, but this interpretation should be made with caution. The absence of a direct effect may reflect more subtle or prolonged interpersonal dynamics, such as the insidious nature of peer conflict or the influence of unmeasured contextual factors. Previous research has shown that lateral violence in nursing tends to affect psychological well-being not through immediate distress, but through internal mechanisms such as diminished self-esteem, impaired coping, or emotional erosion over time [31,32]. It is also possible that this type of violence is not fully captured in a cross-sectional study due to its chronic and often subtle nature. Such forms of aggression may produce cumulative wear and tear that, although less overt, can have significant long-term consequences for mental health. By contrast, violence from patients and supervisors may exert a more immediate psychological toll due to its symbolic weight. Patient aggression often confronts nurses with a distressing role conflict between their caregiving responsibilities and the need to protect themselves—a contradiction that can be ethically troubling, especially when such behaviour is normalized or considered “part of the job”. Meanwhile, supervisor violence undermines vertical trust and perceived organisational justice, thereby deepening emotional strain. Nevertheless, resilience serves as a psychological buffer that not only mitigates but also reshapes the impact of these hostile experiences on nurses’ mental health.

### 4.1. Theoretical and Practical Implications

In summary, the results indicate that exposure to workplace violence exerts a significant and detrimental effect on nurses’ psychological well-being, particularly when such violence originates from users and supervisors. The greater psychological impact of violence from patients and supervisors compared to that from peers may be due to the hierarchical and relational dynamics inherent in healthcare settings. Violence from supervisors undermines perceived organisational justice and safety, whereas patient aggression often evokes feelings of helplessness and role conflict [33]. In contrast, coworker violence, though harmful, may exert its influence through more indirect pathways, such as reduced self-efficacy or social exclusion. Violence from patients and supervisors may exert stronger psychological effects due to the violation of implicit professional expectations. Patient aggression generates role conflict, confronting nurses’ caregiving identity with the need for self-defence. Meanwhile, supervisor violence represents a breach of vertical trust and can undermine perceived organisational justice, intensifying emotional distress [34]. Although psychological resilience plays a mediating role, the estimated indirect effects revealed that its buffering capacity was significant yet partial—especially in the face of patient- and supervisor-related violence.

These findings underscore the protective role of psychological resilience and align with theoretical models, such as Hobfoll’s COR theory, which posits that personal psychological resources buffer individuals against the detrimental effects of stress. The COR theory posits that individuals strive to obtain, retain, and protect their valued resources [19]. Resilience can be conceptualized as a resource reservoir that buffers individuals against resource loss caused by workplace violence. When violence occurs, resilient individuals may activate adaptive coping mechanisms to protect core resources, such as self-esteem, professional identity, or emotional stability. Notably, violence perpetrated by users and supervisors exerted stronger total effects on nurses’ psychological well-being than violence from coworkers, highlighting the importance of tailoring preventive strategies according to the source of aggression. In line with the COR theory, the results suggest that nurses who maintain higher levels of resilience are better equipped to cope with adverse events, such as workplace violence, regardless of its origin. Resilience facilitates more effective emotional regulation and recovery, thereby mitigating the psychological harm associated with traumatic workplace experiences. In hospital environments, where workplace violence is alarmingly frequent, psychological resilience serves as a critical protective mechanism that helps preserve nurses’ mental health.

It is also important to contextualize the effect sizes observed in our mediation models. Although the R^2^ values ranged from 0.06 to 0.11—commonly interpreted as small to moderate—they are consistent with effect sizes typically reported in occupational health and psychosocial research. Given the multifactorial nature of psychological distress and the complex work environments in hospital settings, even modest levels of explained variance can be meaningful. From a practical perspective, small effect sizes may still reflect significant real-world implications when the exposure (e.g., workplace violence) is highly prevalent and the outcomes (e.g., psychological distress) have considerable clinical and organisational impact.

To further explore potential subgroup differences, we conducted additional analyses comparing outcomes across gender, employment contract type (permanent vs. temporary), and work shifts. No statistically significant differences were found by gender or shift schedule. However, we observed small but significant differences based on employment contract. Temporary nurses reported higher psychological resilience and better well-being compared to permanent staff. These differences were not large enough to invalidate the main mediation models, but they suggest that job security might influence how resources, such as resilience, operate under workplace stress. Future research should consider these nuances by exploring interaction effects or stratified analyses.

Although various studies have supported the mediating role of resilience [20,21], others have failed to find such mediation [35]. One possible explanation lies in the nature of resilience as a resource that is most readily elicited in the face of highly stressful or traumatic events [36], such as workplace violence.

From an applied perspective, the findings have relevant implications for personnel management and violence prevention in hospital contexts. Since nurses’ lack of personal resources can amplify the adverse effects of workplace violence on both psychological well-being and the quality of patient care [15], it is essential to develop interventions aimed at enhancing resilience [37].

In this regard, stress management and emotional regulation workshops—including role-playing scenarios, assertive communication training, and techniques drawn from cognitive-behavioural therapy and mindfulness—can offer effective tools for nurses [36,37]. For example, skills training programs that include resilience, coping, problem-solving, and adaptability are often implemented by nurses and psychologists to enhance the psychological competencies of victims of workplace violence. In such cases, resilience increases nurses’ capacity to respond to distressing events related to workplace harassment [38]. Likewise, the group psychoeducational program RISE (Resilience, Insight, Self-Compassion, and Empowerment) has shown positive outcomes in enhancing resilience, self-compassion, and stress management in this population [39]. It is worth noting that these interventions do not necessarily entail high economic costs [36]. Most can be implemented using existing human resources within hospitals, such as clinical psychologists and mental health-trained nursing staff [40,41]. Moreover, they typically require minimal infrastructure and do not depend on expensive materials, which facilitates their integration into existing occupational health or continuing education structures. This type of program has been documented to yield high returns in terms of reducing absenteeism, improving staff retention, and decreasing professional burnout [41.

Many violent incidents against nurses may stem from underlying organisational deficiencies [30]. Therefore, beyond improving nurses’ training to deal with violent behaviour regardless of its origin, one of the main challenges is the effective adaptation of mandatory workplace violence prevention and response protocols within the Spanish healthcare system. Efforts should also be directed toward ensuring the prompt and diligent handling of reports, as well as maintaining psychological support units to help staff cope with the emotional burden of these traumatic events.

Effectively addressing workplace violence requires a dual strategy that combines structural reforms with psychological support programs aimed at strengthening personal resources, particularly resilience. To maximize their effectiveness, these interventions must be both context-sensitive and tailored to the specific source of violence. The present findings reveal that the psychological impact of workplace violence varies depending on its origin, reinforcing the need for differentiated prevention and response strategies. Identifying whether aggression stems from users, coworkers, or supervisors enables the development of more targeted and effective measures to protect healthcare professionals’ well-being and promote safer work environments.

Despite its high prevalence, workplace violence should not be regarded as an inevitable aspect of hospital nursing, but rather as a complex issue requiring specific and multifaceted intervention. Healthcare organisations have a responsibility to ensure safe working environments through the implementation of effective preventive measures and the promotion of an organisational culture grounded in safety, respect, and emotional well-being [40].

Future research would benefit from examining the role of additional personal resources—such as emotional intelligence, self-efficacy, optimism, and coping styles—in the relationship between workplace violence and psychological well-being. Exploring how these factors interact with and complement resilience may offer a more nuanced understanding of the protective mechanisms at play. Furthermore, comparative studies across different hospital departments (e.g., emergency, intensive care, and psychiatry) could identify contextual variations in exposure, impact, and coping strategies. Incorporating organisational-level variables, such as perceived organisational support, leadership style, safety climate, and policy enforcement, could also deepen insight into the structural and cultural factors that influence how violence is experienced and managed. Longitudinal designs are particularly needed to assess causal relationships and the dynamic evolution of resilience and psychological outcomes over time. Additionally, qualitative approaches could help capture the subjective experiences of nurses, providing valuable perspectives that may inform more effective and empathetic intervention programs.

To move toward healthier healthcare organisations, it is essential to strengthen the enforcement of existing regulations, improve mandatory training in workplace violence prevention, and reinforce institutional support. Although legal frameworks exist in Spain to protect healthcare personnel, their implementation remains uneven and limited. Therefore, it is a priority to establish effective protocols and promote an organisational culture of zero tolerance, while also addressing the cultural and structural factors that influence regulatory compliance.

### 4.2. Limitations

Although this study provides valuable insights into the mediating role of resilience in the relationship between workplace violence and psychological well-being, some limitations should be noted. First, its cross-sectional design precludes causal inferences, and longitudinal studies are needed to confirm the stability of the observed effects. Given the cross-sectional nature of this study, causal interpretations should be avoided. Although mediation analyses revealed significant indirect paths, longitudinal designs are required to confirm the directionality and temporal sequence of these relationships.

The use of non-probabilistic purposive sampling in three hospitals from a single region in Spain may limit the generalizability of the results and increase the risk of selection bias. Moreover, although the response rate was relatively high (69.6%), no information is available regarding the reasons for non-participation, which prevents us from completely ruling out the possibility of non-response bias.

Third, the reliance on self-reported data may introduce response bias, despite guaranteed anonymity. In addition, collecting all variables through self-report measures at a single time point raises the possibility of common method variance (CMV), which may artificially inflate the associations between constructs. Although the variables were measured using validated and theoretically distinct instruments, the lack of procedural or statistical controls for CMV is a limitation. Future research should address this issue through design enhancements, such as the use of multiple informants, temporal separation, or objective measures.

Fourth, violence was assessed based on its source (users, colleagues, or supervisors), without differentiating between the various types of aggressive behaviour (verbal, physical, or sexual). However, these dimensions represent qualitatively distinct forms of violence, each with potentially different implications for psychological health. Therefore, future research—particularly studies with larger samples—should delve deeper into the analysis of specific types of violence, including sexual harassment, to better understand their prevalence, impact, and associated mechanisms.

Finally, although key sociodemographic and occupational variables were controlled, other contextual and organisational factors—such as perceived support, workload, or team dynamics—were not assessed and may also play a role in shaping the outcomes.

## 5. Conclusions

This study confirms that psychological resilience acts as a key protective resource for hospital nurses, buffering the negative impact of workplace violence in line with the COR theory. Its main contribution lies in distinguishing the effects of violence by source—users, coworkers, or supervisors—revealing that these sources differ in both intensity and pathways of psychological harm. The findings underscore the need for source-specific interventions. Although user- and supervisor-related violence had stronger effects, peer-related violence was fully mediated by resilience. These distinctions provide a clearer basis for designing targeted preventive and support strategies. From a practical standpoint, this study highlights resilience-building as an essential component of workplace violence prevention. Structured programmes to enhance emotional regulation and coping capacity, combined with institutional measures, are essential for promoting safer and psychologically healthier work environments in hospital settings.

## Figures and Tables

**Table 1:** Means, standard deviations, and correlations among the main variables of this study (*N* = 447).

Variables	Mean	*SD*	1	2	3	4	5	6
1. Age	42.4	10.3	-					
2. User violence	1.50	0.59	−0.14 *	-				
3. Supervisor violence	1.12	0.38	0.02	−0.07	-			
4. Peer violence	1.17	0.40	−0.04	0.14 *	0.44 **	-		
5. Resilience	33.1	3.6	−0.02	−0.16 **	−0.12 *	−0.11 *	-	
6. Psychological well-being	1.7	0.35	0.14 *	0.26 **	0.19 **	0.10 *	−0.27 **	-

* *p* < 0.05; ** *p* < 0.01.

**Table 2:** Results of mediation models.

Model	Outcome Variable	Predictor	*B*	*SE*	95% CI	*p*	R^2^
a	Resilience	User violence	−0.10	0.03	[−0.15, −0.05]	<0.001	0.02
a	Resilience	Supervisor violence	−0.11	0.04	[−0.19, −0.03]	<0.001	0.01
a	Resilience	Peer violence	−0.10	0.04	[−0.18, −0.02]	<0.05	0.01
b + c	Psychological WB	Resilience (User model)	−0.58	0.11	[−0.78, −0.36]	<0.001	0.09
b + c	Psychological WB	User violence	0.36	0.07	[0.23, 0.49]	<0.001	0.11
b + c	Psychological WB	Resilience (Sup. model)	−0.60	0.11	[−0.81, −0.39]	<0.001	0.08
b + c	Psychological WB	Supervisor violence	0.40	0.11	[0.19, 0.61]	<0.001	0.09
b + c	Psychological WB	Resilience (Peer model)	−0.61	0.11	[−0.83, −0.41]	<0.001	0.06
b + c	Psychological WB	Peer violence	0.17	0.10	[−0.02, 0.37]	>0.05	0.07
Indirect	Psychological WB	User violence (via resilience)	**0.058**	0.014	[0.032, 0.089]	<0.001	—
Indirect	Psychological WB	Sup. violence (via resilience)	**0.066**	0.016	[0.038, 0.094]	<0.001	—
Indirect	Psychological WB	Peer violence (via resilience)	**0.061**	0.015	[0.035, 0.087]	<0.001	—

Note: the models were estimated using PROCESS macro (Model 4, v3.1) with 10.000 bootstrapped samples. Indirect effects were calculated as the product of a-path and b-path coefficients; significance was based on 95% bias-corrected confidence intervals that do not include zero. All models were controlled for age, gender, and job tenure. R^2^ is reported for each regression model; indirect effects do not have an independent R^2^ because they are derived from the product of the a- and b-path coefficients. All coefficients are unstandardized. Indirect effects are bootstrapped estimates (10.000 resamples) with 95% bias-corrected confidence intervals. Bold values indicate significance at *p* < 0.05.

## Data Availability

Data are available on request from the authors.

## Public Involvement Statement

There was no public involvement in any aspect of this research.

## Guidelines and Standards Statement

This manuscript was prepared in accordance with the STROBE guidelines for reporting observational studies.

## Abbreviations

The following abbreviations are used in this manuscript:

COR	Conservation of Resources
